# Mercy Hospital

**Published:** 1879-01

**Authors:** F. E. Waxham


### Clinical Reports.

---

### ARTICLE V.

### MERCY HOSPITAL.

REPORTED BY F. E. WAXHAM, M.D.

CASE I—*Stricture of Urethra.*

The patient entered the hospital Nov. 2d, with a very tight stricture. This had existed for twelve years. In 1862 he had an attack of gonorrhœa followed by gleet. Has had complete retention of urine several times, and was relieved only by tapping the bladder above the pubis. On entering the hospital, the stricture was so tight that nothing could be passed through it, although a No. 1 instrument entered a short distance. From day to day the attempt was repeated to pass the stricture, but unsuccessfully. On Nov. 6th complete retention occurred. After many trials and a great deal of patient labor, a very small filiform bougie was passed through the stricture into the bladder. This dilated his urethra so that he was able to pass water. Shortly after, he suffered a very severe urethral chill, followed not by fever but by a very copious perspiration.

Nov. 8th, complete retention again occurred. Patient suffered greatly from spasmodic contraction of the bladder. The pain and spasm were controlled by opiates and monobromated camphor, the latter in two decigram doses repeated every hour. Under this treatment he soon slept soundly, and passed his urine involuntarily while in this condition.

Nov. 11th. Anæsthetized, and internal urethrotomy performed by anterior and lateral incisions. A very small filiform bougie was first passed by the stricture and the free extremity attached to a staff, the former instrument curling up in the bladder and acting as a guide to the staff which followed it. The urethretome was then passed down the groove in the staff, first anteriorly and then laterally, thus severing the stricture. A No. 10 sound was introduced into the bladder, followed by a No. 12 and No. 16. Shortly after the operation the patient suffered a very severe urethral chill. His temperature fell to 95½. He, however, had no repetition of these symptoms, and was discharged, cured, Nov. 20th, able to pass a full stream of water.

CASE II—*Stricture of Urethra with Perineal Fistula.*

Stephen Wright. Age 55. Entered the hospital with the following history: About five years since, while carrying a heavy weight on his shoulder, he fell across a well-curb, injuring his perineum very severely. He has suffered from gradually contracting stricture since that time. Last summer he was able to pass his water only drop by drop. In July infiltration of urine into the perineum and scrotum occurred. This being mistaken for erysipelas, a great amount of sloughing of perineum and scrotum took place before free incisions were made. The damage has gradually been repaired, but a small urinary fistula has been the result.

Nov. 11th. As it was impossible to cure the fistula as long as the stricture existed, urethrotomy was performed, as in the former case. A few hours after the operation this patient also suffered a very severe urethral chill.

Dec. 20th. Stricture has yielded completely, and fistula nearly healed; general health excellent.

CASE III—*Artificial Anus.*

Mrs. C.— Age 35. A year and a half ago the patient suffered from a strangulated inguinal hernia. Dr. Andrews being called five days after the strangulation occurred, found her in a very feeble condition. Although little hope of saving her life was entertained, she was operated upon, and six inches of dead intestine were removed. The healthy edges of the intestine were stitched to the opening, making an artificial anus. The patient made a gradual recovery.

On entering the hospital last April, she was pale and very anæmic. Nearly all the passages from the bowels were through the artificial opening. Occasionally a very small passage would occur through the natural canal, showing that the lower portions of the bowels were still patulous. The first operation consisted in placing a clamp upon the septum of the intestine at the opening, thus removing it by gradual suppuration. Several operations were performed for the closure of the opening, each time freshening the edges of the wound and bringing them together by silver wire sutures, tightened by preforated shot or a lead button. This patient returned home in August with the opening nearly closed, it being no larger than a crow's quill. The patient returned Oct. 30th to have this small opening closed although there was but very little discharge from it.

Nov. 11th. The edges of the opening were pared off and incisions were made through the integument and cellular tissue about an inch from the wound on each side, that the freshened surfaces might be approximated. An opening was also made from the lower incision under the integument and areolar tissue to the opening in the intestinal canal, and a drainage tube inserted in order to give the old opening a better opportunity to heal. The freshened edges of the opening were then brought together by silver sutures. The operation proved successful.

Dec. 9th. Again anæsthetized and the whole surface of the old cicatrix removed and a flap two inches square taken from below on the thigh and layed over this. The edges were united by sutures all around, excepting on one side, where an opening was left communicating with the intestine. A drainage tube was inserted in this opening under the flap.

Dec. 14th. Patient suffered severely for several days, but at present date is doing well.

CASE IV—*Enlargement of Prostate.*

Peter Peterson. Age 59. Entered the hospital Nov. 7th, with the following history: He had always been healthy until a year and a half ago, when profuse hæmorrhage occurred from the urethra, lasting two days and prostrating him very much. Hæmorrhage recurred once or twice between that time and June 17th, 1878, at which date he bled until death seemed at hand. He has had increasing frequency of micturition until the present time, when he frequently can not retain his urine longer than ten minutes.

Nov. 9th. Made an incomplete examination for stone. Found the prostate much enlarged, forming a tumor which nearly occluded the rectum.

Nov. 11th, the patient was anæsthetized and a thorough examination made. No calculus was discovered. The bladder was found contracted and its walls thick and grisly. The prostate was enormously enlarged, but it was thought to be probably a simple enlargement without possessing any malignant characters.

Nov. 29th. Left, somewhat improved under the effect of astringent injections.

